# PPAR*δ* Agonism for the Treatment of Obesity and Associated Disorders: Challenges and Opportunities

**DOI:** 10.1155/2008/125387

**Published:** 2008-10-29

**Authors:** Mylène Perreault, David V. Erbe, James F. Tobin

**Affiliations:** Department of Cardiovascular and Metabolic Diseases, Wyeth Research, 200 Cambridge Park Drive, Cambridge, MA 02140, USA

## Abstract

The prevalence of obesity in the USA and worldwide has reached epidemic proportions during the last two decades. Drugs currently available for the treatment of obesity provide no more than 5% placebo-adjusted weight loss and are associated with undesirable side effects. Peroxisome proliferator-activated receptor (PPAR) modulators offer potential benefits for the treatment of obesity and its associated complications but their development has been complicated by biological, technical, and regulatory challenges. Despite significant challenges, PPAR modulators are attractive targets for the treatment of obesity and could offer a viable alternative to the millions of patients who fail to lose weight following rigorous dieting and exercise protocols. In addition, PPAR modulators have the potential-added benefit of ameliorating the associated comorbidities.

## 1. INTRODUCTION

During the last two decades, the incidence of obesity has tripled in developing countries, with more than 1.1 billion adults overweight worldwide and 312 million of them obese [1]. Obesity has been causally linked to the development of certain forms of cancer, cardiovascular disease, sleep apnea, psychiatric disorder, and type 2 diabetes (T2D) [2–6]. In parallel to the growing rate of obesity, the incidence of T2D has also dramatically increased worldwide, and it is believed that more than 366 million people will become diabetic by 2030 [1]. Recent findings point toward visceral adiposity as a causal link for the development of obesity-induced T2D. Visceral adipose tissue (VAT) is metabolically more active than subcutaneous adipose tissue and secretes a number of adipokines [7, 8]. The surgical removal of as little as 0.6 kg (0.8% total fat) of VAT significantly ameliorated insulin sensitivity in obese patients [9], whereas removal of large amounts of subcutaneous adipose tissue did not [10]. These findings suggested that adipose tissue can no longer be considered an inert tissue strictly involved in lipid storage but is rather an endocrine organ capable of regulating several aspects of metabolism.

Currently, a few drugs are approved for the long-term treatment of obesity but their efficacy is limited. Both sibutramine (Meridia) and orlistat (Xenical) have been available for approximately 10 years for the treatment of obesity but these agents only induce modest one-year weight loss of 4.2% and 2.9%, respectively [11]. More recently, the cannabinoid receptor-1(CB1) inverse agonist, rimonabant, has been associated with significant weight loss in several global clinical trials, including RIO-Europe, RIO-Lipids, RIO-Diabetes, and RIO-North America [11]. Although rimonabant induced an approximately 5% placebo-adjusted weight reduction in those studies, it was not approved in the USA principally due to neurological and psychiatric safety concerns [12]. Recently, the antidiabetic GLP-1 agonist exenatide (Byetta) was shown to significantly reduce mean body weight (−4.7%) from baseline after 2 years of treatment [13]. This drug offers benefits over existing therapies as it combines weight loss and improvements in glycosylated hemoglobin (HbA1c), liver function, and blood pressure [13]. Side effects including nausea and vomiting have been noted [14] and rare cases of acute pancreatitis have been reported [15]. However, exenatide is currently not approved for the treatment of obesity.

With no more than 5% placebo-adjusted weight loss observed with currently available treatments, more drugs are thus needed to provide patients with safe and efficacious therapeutic options. Recent findings have suggested that peroxisome proliferator-activated receptor (PPAR) delta agonists could induce body weight loss by increasing energy expenditure and fatty acid oxidation [16]. In fact, PPAR*δ* agonism either alone or in combination with agonism of PPAR*α* and/or PPAR*γ* could not only induce body weight loss but could also potentially improve other metabolic parameters including insulin sensitivity and lipid profiles, thus producing benefits associated with the comorbidities of the obese state.

## 2. MECHANISM OF ACTION OF PPARs

PPARs belong to the nuclear receptor family of transcription factors and are master regulators of genes involved in glucose and lipid metabolism. The PPARs are modular in nature, containing an amino-terminal ligand-independent AF1 transactivation domain, a DNA binding domain containing two zinc finger motifs, and a carboxy-terminal domain that contains a dimerization domain and a ligand-dependent AF-2 transactivation domain [17]. There are three members of the PPAR family, PPAR*α*, PPAR*γ*, and PPAR*δ* [18]. They are 60–80% conserved in their DNA and ligand-binding domains and are activated by a number of natural and synthetic ligands, including eicosanoids, free fatty acids (FFA), lipid lowering drugs (fibrates), and the insulin sensitizers (thiazolidinediones). PPARs heterodimerize with the retinoic X receptor (RXR) and bindspecific promoter sequences termed PPAR responsive elements (PPREs) in their target genes. In the absence of a ligand, PPARs form complexes with corepressors such as NcoR, RIP140, and SMRT [19–21], which repress transcription through the recruitment of histone deacetylases. Ligand binding induces a conformational change that results in the dissociation of corepressors and the recruitment of coactivators, such as PPAR*γ* coactivator-1 (PGC-1), mediator complex, P300/CBP [22–24]. The specificity of complexes formed between the receptor and corepressors or coactivators appears to be determined by cell type, conformational change induced by the ligand, and sequence of the DNA binding element. This level of complexity allows fine-tuning of the physiological response and explains the variability of gene expression changes when a receptor is activated by different ligands.

### 2.1. PPAR*γ*

PPAR*γ* is the molecular target of thiazolidinediones (TZDs) and has been well characterized in obese diabetic rodent models and in diabetic patients. It is primarily expressed in adipocytes, where it plays a pivotal role in the regulation of adipocyte differentiation and lipid storage. Treatment of diabetic animals or patients with TZDs increases subcutaneous fat mass area by inducing the number of small insulin sensitive adipocytes in subcutaneous fat [25–28], while decreasing visceral fat mass [29, 30]. This effect of TZDs most likely contributes to the improvement of insulin sensitivity, although a direct effect of PPAR*γ* on skeletal muscle, liver, and macrophages has also been demonstrated. For example, it has been reported that the insulin sensitizing effect of rosiglitazone was attenuated in macrophage-specific PPAR*γ* knockout mice, suggesting that macrophages are important for TZD-induced insulin sensitivity [31]. TZD treatment has also been associated with significant improvement of skeletal muscle, liver, and adipose tissue insulin signaling in both rodents and humans [30, 32, 33]. Additionally, PPAR*γ* agonists increase the expression of adiponectin, which decreases hepatic glucose production through activation of AMP-activated protein kinase (AMPK) [34] and could thus contribute to TZD-induced improvement in insulin sensitivity.

### 2.2. PPAR*α*

PPAR*α* is predominantly expressed in highly oxidative tissues, including liver, skeletal muscle, brown adipose tissue, and heart [35, 36] and is the target of the fibrate class of drugs used for the treatment of dyslipidemia. Fibrates decrease triglyceride levels in both rodents and humans by depleting the pool of free fatty acids (FFAs) through peroxisomal and mitochondrial fatty acid oxidation. PPAR*α* activation has been associated with increased expression of genes involved in all steps of fatty acid utilization, including the breakdown of triglyceride particles to free fatty acids by lipoprotein lipase (LPL), the transport of free fatty acids inside the cells by fatty acid transport proteins (FATPs), and the peroxisomal and mitochondrial *β*-oxydation by activation of acyl CoA oxydase (ACO) and medium chain acyl CoA decarboxylase (MCAD). In addition, PPAR*α* is involved in the reverse cholesterol transport pathway and contributes to HDL production in humans by increasing the expression of the major constituent of high-density lipoprotein (HDL) cholesterol, apolipoprotein A1 (ApoA1) [37], as well as increasing the ATP-binding cassette transporter A1 (ABCA1) [37].

Recently, PPAR*α* has also been implicated in the regulation of body weight through appetite suppression. In rodents, PPAR*α* agonists, including WY-14643, fenofibrate, and oleoylethanolamide (OEA), have been reported to induce body weight loss in a PPAR*α*-specific manner [38, 39]. One of the proposed mechanisms involves stimulation of specific regions of the brain controlling satiety through [40] vagal nerve activation. Interestingly, PPAR*α* agonists can no longer induce appetite suppression in rats in which the vagal nerve has been severed [38]. It is also possible that PPAR*α* induces appetite suppression through other mechanisms, including FGF-21 secretion [41–43] or increased fatty acid oxidation and production of ketone bodies [44, 45].

### 2.3. PPAR*δ*

PPAR*δ* has recently been characterized in rodents and humans and is more widely distributed than its two counterparts with high level of expression in skeletal muscle, heart, kidney, adipose tissue, liver, and macrophages [46]. The physiological role of PPAR*δ* has been elucidated not only through genetic approaches, but also by pharmacological activation of the receptor with specific agonists, including GW501516. A major role for PPAR*δ* in body weight regulation has been reported in mice overexpressing a constitutively active form of PPAR*δ* in adipose tissue. These mice are resistant to diet-induced obesity (DIO) and are protected against the development of genetically induced obesity through induction of energy expenditure via mitochondrial fatty acid oxidation and energy uncoupling [16]. It is also possible that PPAR*δ* induces body weight loss by centrally regulating appetite suppression since PPAR*δ* was shown to mediate the hyperphagic response following focal cerebral ischemia [47].

In addition to inducing body weight loss, PPAR*δ* activation has been shown to improve lipid profile by depleting the pool of fatty acids through mitochondrial fatty acid oxidation [16, 48] and by increasing reverse cholesterol transport [49]. Two-week treatment of healthy volunteers with GW501516 significantly increased HDLc and decreased triglycerides as compared to placebo-treated subjects [50]. PPAR*δ* expression is upregulated in skeletal muscle of T2D subjects and high-fat fed rats after bouts of exercise [46, 51–54] and may play a role in providing a continuous source of energy to support the increasing energy demand. Consistent with this hypothesis, skeletal muscle overexpression of PPAR*δ* was associated with significant muscle-type switching from glycolytic to oxidative fibers, further supporting its role in lipid oxidation [55]. Interestingly, skeletal muscle PPAR*δ* transgenic mice were more resistant to fatigue after intense exercise [55], suggesting a PPAR*δ*-induced switch in substrate utilization that helped maintain a higher level of energy demand.

In addition to inducing weight loss and improving lipid profiles, PPAR*δ* agonism has the potential to improve insulin sensitivity and glucose metabolism [46, 56]. Recently, this has been shown to include a crucial role for PPAR*δ* expressed in tissue macrophages [57, 58]. Activation of resident macrophages in metabolic tissues toward the inflammatory (M1) phenotype appears to play a role in insulin resistance as a result of obesity. However, through production of Th2 cytokines, both adipocytes and hepatocytes are able to induce PPAR*δ* expression in resident macrophages leading to their alternative activation toward the anti-inflammatory M2 phenotype. This then results in increased insulin sensitivity in these tissues, and could underlie some of the improvements in glucose metabolism seen in PPAR*δ*-treated animals.

## 3. CHALLENGES TO THE DEVELOPMENT OF PPAR*δ* MODULATORS

Following the publication of several high-profile studies implicating PPAR*δ* as a potential target for obesity and associated metabolic disorders, multiple pharmaceutical companies have initiated drug discovery efforts to identify specific PPAR*δ*, PPAR*α*/*δ*, PPAR*γ*/*δ*, and pan PPAR modulators. However, development of these PPAR agonists faces significant challenges including major differences in PPAR biology between rodents and humans, difficulties in predicting in vivo efficacy from simple in vitro models of activity, toxicological findings arising from weight reduction through targeting *β*-oxidation of fatty acids, and safety requirements unique to PPAR biology and drug development.

### 3.1. Differences in PPAR pharmacology between rodents and humans

Despite very high homology between human and rodent PPAR receptors, major biological and physiological differences exist between these species. One clear and well-understood example relates to peroxisome proliferation and liver enlargement with PPAR*α* agonists. In rodents, but not humans, PPAR*α* activation leads to hepatic peroxisome proliferation associated with liver enlargement and cell necrosis. One potential biological explanation for these differences is that hepatic PPAR*α* expression is approximately 10 times higher in rodents versus humans [59].

Similarly, it has been reported that PPAR*δ* pharmacology differs between higher species and rodents. Contradicting reports suggest that pharmacological PPAR*δ* activation inconsistently regulates body weight [55, 60, 61] and cholesterol or triglyceride levels in rodents [55, 60–62]. However, significant elevation of HDL cholesterol and/or reduction of LDL cholesterol and triglyceride have been reported in obese rhesus monkeys and humans after chronic PPAR*δ* agonist administration [50, 63]. One potential explanation may reside in the observation that the rodent ApoA-1 promoter differs from the human promoter by 3 nucleotides, resulting in a nonfunctional PPRE site [64, 65]. Interestingly, PPAR*δ* agonists have been shown to decrease triglyceride and increase HDL cholesterol in humanized ApoA-1 transgenic mice [66] suggesting that mice can positively respond to PPAR*δ* activation once provided with a functional ApoA1 gene. Overall, these species differences could make the observation of PPAR*δ* driven outcomes more difficult in rodents, and thus complicate the development of PPAR*δ* modulators.

### 3.2. Complexity induced by ligand-specific effects

Traditionally, the potency and affinity of PPAR modulators have been determined using multiple assays, including displacement of radiolabeled compound, displacement and recruitment of corepressors and coactivators, and cell-based transcriptional activation assays. Often, the in vitro potency in binding and transcriptional activation assays correlates with in vivo efficacy, a notable example is the hypoglycemic effects of TZDs. TZDs with more potent in vitro activities are often more active in rodent models [67, 68]. In some cases, these correlations do not hold, reflecting a more complex relationship between simple in vitro measures and in vivo pharmacology that is best understood in the context of the selective PPAR modulator (SPPARM) hypothesis. The SPPARM concept emerged to describe the complete spectrum of PPAR conformation/activation states leading to each specific biological response. This model stipulates that PPAR modulators turn on and off specific genes by recruiting or releasing a complex assortment of coactivators and corepressors. The coactivators and corepressors are likely tissue specific and different modulators are believed to recruit different sets of proteins leading to various degree of gene activation/repression. This level of complexity provides a potential strategy to eliminate the known liabilities associated with PPAR activation by selecting modulators that are recruiting/releasing specific sets of coactivators and corepressors. However, this complexity also complicates the development of PPAR agonists because species differences can exist concerning coactivator expression and recruitment, tissue specificity and availability.

Consistent with the SPPARM hypothesis, Berger et al. reported that PPAR*γ*/*δ* agonists L-165461 and L-783483 had similar binding affinities and transcriptional activities against PPAR*γ* and PPAR*δ* while the glucose lowering effect was more pronounced with L-783483 in db/db mice [67]. We have observed that PPAR pan modulators with similar profiles in transcriptional cell-based assays can yield significantly different in vivo activities (Table 1) despite similar pharmacokinetic properties. These differences likely stem from differential interactions with the ligand-binding pocket of each PPAR resulting in modulator-specific differential recruitment of coactivators/corepressors.

### 3.3. Challenges associated with the development of weight-reducing agents targeting oxidation of fatty acids

It is well understood that body weight is physiologically regulated through two major mechanisms: food intake and energy expenditure. Energy expenditure can be positively or negatively modulated by regulating metabolic rate, body temperature, or level of physical activity. Targeting metabolic rate through oxidation of free fatty acids and/or energy uncoupling are effective ways to induce body weight loss. For example, it has been shown that targeted deletion of acetyl-CoA carboxylase-2 (ACC2), an enzyme responsible for the synthesis of malonyl-CoA, is associated with approximately 10–20% body weight loss caused by significant increase in total energy expenditure, with no change [71] or increased food consumption [72]. The increased energy expenditure was mostly explained by a significant increase in fatty acid oxidation and not an elevation of the level of physical activity [71, 72]. Similarly, transgenic mice with muscle-specific activation of PPAR*α* are resistant to diet-induced obesity despite consuming similar amounts of food compared to nontransgenic mice [73]. As expected, the rate of palmitate oxidation and the level of expression of genes regulating mitochondrial and peroxisomal *β*-oxidation were increased in muscle-specific PPAR*α* transgenic mice [73]. Overall, these studies provided convincing evidence that specifically targeting oxidation of fatty acids is a relevant mechanism to induce body weight loss.

PPAR*δ* is known to influence all of the parameters regulating energy expenditure by increasing the transport and oxidation of free fatty acids in adipose tissue and skeletal muscle, increasing thermogenesis and energy uncoupling in adipose tissue and inducing muscle fiber-type switching to promote endurance and resistance to fatigue. Recently, PPAR*δ* has been suggested as a potential target for body weight loss because of its role in fatty acid oxidation. In mouse and human skeletal muscle, PPAR*δ* activation increases the expression of several genes involved in mitochondrial oxidation of free fatty acids, including carnitine palmitoyltransferase-1 (CPT-1), medium chain acyl-CoA dehydrogenase (MCAD), long chain acyl-CoA dehydrogenase (LCAD), and PGC-1*α* [16, 48, 55, 74] and its pharmacological activation has been associated with body weight loss in rodents [48, 55].

Currently, controversies exist as to whether an increased rate of fatty acid oxidation is beneficial. Numerous studies have been published demonstrating that an elevated rate of *β*-oxidation is beneficial because it depletes the pool of fatty acids required to synthesize triglyceride particles, prevents accumulation of fatty acid metabolites in skeletal muscle and other nonfat tissues, and induces body weight loss. However, it has been shown that sustained level of fatty acid oxidation, through long-term fibrate treatment or PPAR*α* overexpression, is associated with skeletal and heart muscle degeneration [75], cardiomyopathy [76, 77], and insulin resistance [73] in mice. Consistent with these reports, we have previously observed that pan PPAR activation induced significant levels of skeletal muscle degeneration and liver vacuolation and necrosis in mice, but these observations were completely absent in PPAR*α* knockout animals (M. Perreault et al., unpublished observations). Collectively, these results suggest that sustained levels of fatty acid oxidation through PPAR*α* and potentially PPAR*δ* could significantly improve some metabolic parameters but with potential deleterious effects on muscle and liver.

It has been proposed that the detrimental effects of sustained fatty acid oxidation result from the production of reactive oxygen species produced during peroxisomal and mitochondrial oxidation of fatty acids. The first and rate-limiting step of peroxisomal *β*-oxidation, ACO, generates hydrogen peroxide during oxidation of acyl-CoAs. It has been reported that hydrogen peroxide levels are significantly increased in the heart of cardiac-specific PPAR*α* transgenic mice and this effect is exacerbated on a high-fat diet, where the substrates for fatty acid oxidation are more abundant [76]. Similarly, oxidation of fatty acids through mitochondrial *β*-oxidation has been associated with an increased production of reactive oxygen species (ROSs) as long-chain fatty acids provide reducing equivalents that fuel the electron transport chain. 

While the role of PPAR*α* in skeletal and cardiac muscle degeneration has been established in rodents, the effect of PPAR*δ* activation on these parameters is less clear. It has been reported that PPAR*δ* activates distinct metabolic programs in the mouse heart as compared to PPAR*α*, leading to cardioprotection in the setting of myocardial ischemia/reperfusion injury [77]. The exact mechanism for the cardioprotection is currently unknown, but in contrast to PPAR*α*, PPAR*δ* overexpression induced cardiac glucose oxidation as opposed to fatty acid oxidation [77]. Normal hearts exhibit substrate flexibility by switching between lipid and glucose to match the metabolic state. However, diabetic hearts mostly rely on fatty acids, leading to excessive rates of myocardial fatty acid oxidation concomitant with reduced glucose oxidation. In the heart of cardiac-specific PPAR*α* transgenic mice, genes involved in fatty acid uptake, lipogenesis, and triglyceride synthesis (fatty acid transport protein (FATP), CD36, glycerol-3-phosphate acyltransferase (GPAT), acyl-CoA synthetase (ACS), fatty acid synthase (FAS), microsomal triglyceride transfer protein (MTP)) were significantly elevated while genes involved in glucose metabolism (glucose transporter 4 (GLUT4), phosphofructokinase (PFK)) were not. In contrast, the expression of fatty acid transport and esterification genes was unchanged in cardiac-specific PPAR*δ* transgenic mice while genes regulating glucose metabolism were significantly elevated. These results further contribute to the hypothesis suggesting that increased and sustained rate of fatty acid oxidation could be detrimental for cardiac functions and suggest that this mechanism is PPAR*α*-specific. Furthermore, it has been reported that PPAR*δ* activation is associated with elevated expression of catalase [78], an enzyme responsible for hydrogen peroxide degradation potentially providing an additional protective effect.

Recently, the concept of incomplete fatty acid oxidation has emerged as another potential explanation for fatty acid oxidation-induced metabolic disturbances. It was proposed that obesity results in an excessive fatty acid load on mitochondria causing accumulation of incompletely oxidized intermediates, including acylcarnitine esters, while decreasing levels of metabolites of the tricarboxylic acid (TCA) cycle [79]. The exact mechanism leading to metabolic disturbances is still unknown and whether acylcarnitine esters directly induce insulin resistance and metabolic disorders remains to be determined.

### 3.4. Safety study requirements unique to PPAR biology and drug development

PPAR gamma (TZDs) and alpha (fibrates) agonists have been used for many years to treat type 2 diabetic and dyslipidemic patients, respectively. TZDs (Avandia and Actos) are very well characterized in rodents and humans and result in significant improvements in insulin sensitivity and glycemic control but are associated with increases in body weight and fluid retention that can exacerbate congestive heart failure. The liabilities associated with PPAR*γ* agonists are observed in a small but significant number of patients and are very closely related to their efficacy, as reduction in HbA1c is directly correlated to body weight gain [80]. PPAR*γ*-associated weight gain and fluid retention liabilities are well understood and can be reversed by discontinuing drug administration or by treating with diuretics.

Recently, the PPAR*γ* activator rosiglitazone has been suggested to directly affect cardiac function independent from its effects on fluid retention. A number of meta-analyses concluded that rosiglitazone significantly increased the rates of myocardial infarction and death by cardiovascular causes [81, 82]. However, controversies exist as several recent studies refuting those results have been published [83, 84]. Interestingly, a significant lower risk of death, myocardial infarction and stroke was observed with pioglitazone (Actos) [85], suggesting a difference between rosiglitazone and pioglitazone in relation to cardiovascular effects. Pioglitazone has been previously shown to improve lipid profile (triglyceride, LDL and HDL cholesterol) by weakly activating PPAR*α*, while rosiglitazone seems to worsen these parameters which could contribute to the deleterious effects on cardiac functions [86–88]. The findings from these meta-analyses will have to be confirmed with cardiovascular outcome studies, as the clinical trials included in these analyses were not originally designed to evaluate cardiovascular endpoints. The RECORD study is a clinical trial evaluating the effects of rosiglitazone in approximately 2000 patients to specifically address cardiovascular effects as primary outcomes. The interim analysis indicated no significant increased myocardial infarction and death with rosiglitazone, however, the authors admitted that their study might be underpowered due to lower cardiovascular events in this patient population and that the analysis was performed before completion of the study [89–92].

Whether the cardiovascular effects of rosiglitazone and pioglitazone are confirmed or not with clinical outcome studies, these recent findings have changed the way regulatory agencies review and approve drugs and protein therapeutics for the treatment of T2D. Earlier this year, the FDA published their draft guidance for the development of new diabetes therapeutics. The guidance affirms that in the absence of cardiovascular signal, long-term cardiovascular studies could be conducted postapproval in a reasonable timeframe [93]. However, large outcome trials should be conducted prior to submission of regulatory dossiers for drugs that show nonclinical or clinical evidence of increasing cardiovascular risk [93].

In addition to the cardiovascular effects, multiple PPAR modulators have been discontinued over the last several years due to carcinogenicity findings in rodents. Because of the prevalence of positive carcinogenicity findings with PPAR agonists and the lack of complete understanding of PPAR-induced tumor development, 2-year carcinogenicity studies in mice and rats are now required before clinical trials longer than 6 months in duration can be initiated [93, 94]. Moreover, PPAR*γ* activation has recently been linked to an increased risk of fracture in humans [95–97]. It has been proposed that PPAR*γ* activators could have a direct effect on osteoblastogenesis [98, 99] and osteoclastogenesis [100] in rodents, but further clinical studies will be necessary to understand if these mechanisms are also involved in TZDs-induced bone loss in humans. Despite an increased risk of fracture with PPAR*γ* activators in humans, the role of other PPAR isoforms on bone formation and resorption is less clear. The presence of PPAR*δ* both at protein and mRNA levels has been reported in rat bone tissue sections, preosteoblasts, rodent and human osteoblastic cell lines as well as rabbit osteoclasts [101–104], while PPAR*α* has been found in preosteoblasts, chondrocytes, and human peripheral blood mononuclear cell (PBMC)-derived osteoclasts [101]. The role of PPAR*α* and PPAR*δ* in bone formation and resorption needs to be further determined.

## 4. SUMMARY AND PERSPECTIVES

Obesity and associated disorders are serious diseases affecting millions of people worldwide. The therapeutic options currently offered are providing limited efficacy and are coupled with several serious side effects. Despite significant challenges associated with their development, PPAR modulators are potential new obesity therapies that could offer not only weight management opportunities but also amelioration of the associated disorders by correcting the causes of insulin resistance and dyslipidemia. Molecules targeting PPAR*δ*, alone or in combination with other PPARs, offer significant advantages as PPAR*δ* seems to have beneficial cardioprotective effects in rodents by modulating fuel utilization in addition to its anti-inflammatory and lipid effects. Whether or not such weight-modulating therapies will be relevant to humans and ultimately approvable by regulatory agencies remain to be determined.

## Figures and Tables

**Table 1:** Compounds with similar in vitro profiles induced different in vivo efficacy.

Compound	In vitro potentcy (*μ*M)			Weight loss	Insulin (% reduction)	Adiponectin (fold increase)

	*α*	*γ*	*δ*
**1** [69, 70]	6.0	0.1	5.0	Weight neutral	30%	2.1
**2** [69, 70]	3.0	0.1	1.0	14%	80%	3.2
